# Development and Preliminary Evaluation of a Spray Deposition Sensing System for Improving Pesticide Application

**DOI:** 10.3390/s151229898

**Published:** 2015-12-17

**Authors:** Melissa A. Kesterson, Joe D. Luck, Michael P. Sama

**Affiliations:** 1 Department of Mechanical and Materials Engineering, University of Nebraska-Lincoln, Lincoln, NE 68583, USA; melissa.kesterson@yahoo.com; 2 Department of Biological Systems Engineering, University of Nebraska-Lincoln, Lincoln, NE 68583, USA; 3 Department of Biosystems and Agricultural Engineering, University of Kentucky, Lexington, KY 40546, USA; michael.sama@uky.edu

**Keywords:** spray deposition, pesticides, droplet spectra, wireless data acquisition

## Abstract

An electronic, resistance-based sensor array and data acquisition system was developed to measure spray deposition from hydraulic nozzles. The sensor surface consisted of several parallel tin plated copper traces of varying widths with varying gap widths. The system contained an embedded microprocessor to monitor output voltage corresponding to spray deposition every second. In addition, a wireless module was used to transmit the voltage values to a remote laptop. Tests were conducted in two stages to evaluate the performance of the sensor array in an attempt to quantify the spray deposition. Initial tests utilized manual droplet placement on the sensor surface to determine the effects of temperature and droplet size on voltage output. Secondary testing utilized a spray chamber to pass nozzles at different speeds above the sensor surface to determine if output varied based on different application rates or spray droplet classification. Results from this preliminary analysis indicated that manual droplets of 5 and 10 μL resulted in significantly different values from the sensors while temperature did not consistently affect output. Spray chamber test results indicated that different application rates and droplet sizes could be determined using the sensor array.

## 1. Introduction

Pesticide application errors are a time consuming and expensive challenge that producers face throughout the world. A major concern with pesticide application today is related to off-target movement of spray particles (*i.e.*, spray drift); which has become a focus area at the United States Environmental Protection Agency. Essentially, smaller particles are susceptible to drift and can expose people, wildlife, and the environment to pesticide residues which can cause health and environmental effects as well as property damage. To date, there have been many testing procedures conducted to measure spray droplet and spray deposition. One of the most common methods includes using water sensitive paper (WSP) and traditional optical techniques that involve analyzing images of droplets from spray nozzles. One drawback of WSP is that the paper can only be used when the application volume is low enough the overlap stains do not saturate the entire paper. More accurate methods have involved using fluorometry [1] and colorimetry [2]. In laboratory settings, it is common to use laser particle analyzers for measuring spray patterns from nozzles to determine droplet size distributions and have seen some in-field use [3]; however these systems can be expensive. Other methods for measuring spray droplets and spray deposition (e.g., water sensitive paper and string collectors) can be time consuming, expensive, and lack sufficient accuracy. Researchers and manufacturers would benefit from an automated system that could estimate droplet size distributions among various droplet sizes.

Previous studies have been conducted in an attempt to develop and evaluate electronic methods for detecting particles from hydraulic nozzles [4] developed an electronic sensor that measured voltage output from fixed parallel traces to estimate spray deposition on the sensor surface. Results indicated that output voltage was proportional to the amount of spray deposited on the surface; however reliability was questionable for the system. A similar system using parallel traces to measure output voltage was tested for measuring humidity levels in controlled environments [5]. A limitation of both systems was that fixed trace and gap widths were used for the surface of the sensing systems. The ability to use an array of these types of sensors to simultaneously monitor spray deposition should be investigated. [6] developed a digital system to monitor spray particle deposition. Further development was needed to improve resolution of the sensing pads and the need for wireless data acquisition (DAQ) was also expressed. While some recently developments have been made to improve the analysis time using WSP including image processing [7] and smartphone, web-based tools [8], little effort has been made to further the measurement of spray application or droplet sizes from hydraulic nozzles using an electronic sensing platform. Further research is needed to stimulate advances and the development of technologies related to spray application monitoring.

The goal of this study was to conduct a preliminary evaluation on the performance of a wireless, electronic sensor array for its potential to replace traditional WSP-based methods for assessing droplet spectra and nozzle application rates. Two novel aspects of this system were the integration of multiple trace and gap widths on the sensing surface and the use of a wireless module for data transfer. Specific objectives for this study were to: (1) test the wireless DAQ system for monitoring sensor output; (2) determine if sensor array performance was consistent with respect to orientation of droplets on sensor, temperature and droplet sizes; and (3) evaluate sensor array output for different application rates and droplet spectra classification at constant nozzle pressures.

## 2. Materials and Methods

### 2.1. Sensor Array and Data Acquisition Development

**Figure 1:**
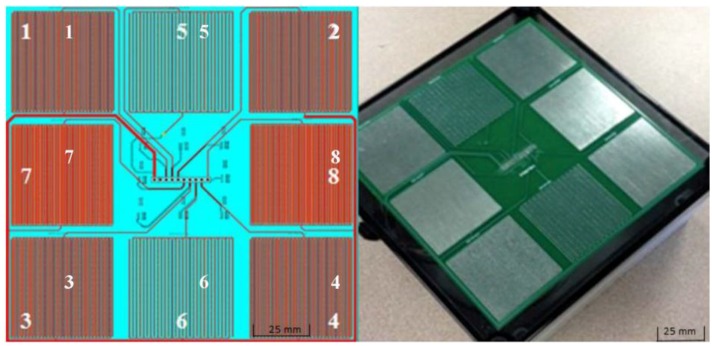
Design schematic with sensor ID numbers (**left**) and photo (**right**) of spray deposition sensor array used during testing.

The sensing platform developed consisted of a resistance-based printed circuit board and wireless DAQ hardware using an embedded microprocessor. Each sensor was comprised of a configuration of parallel traces with gaps fabricated from tin plated copper material during the board printing process. A design schematic and photo of the sensor array is shown in Figure 1. Three different sensor configurations were printed on the sensor array circuit board. Sensors 7 and 8 represented the smallest trace and gap widths; both trace and gap widths were increased for Sensors 1 through 4; gap widths were then increased for the design of Sensors 5 and 6. A summary of the trace and gap widths with the resulting spacing is shown in Table 1. As shown in Table 1, each sensor design was replicated at least once in the final sensor array used for testing.

A common 9 VDC input was supplied to each of the sensors as was a common ground via internal circuitry on the printed board. A variable resistor was soldered between each sensor and ground, essentially creating a voltage divider for the eight separate circuits. The resistance setting for each sensor plate output was calibrated to produce 5 VDC while each sensor was saturated with water. The set value for each variable resistor was monitored with a digital multimeter during this calibration process. A summary of the resistance settings for the three sensor configurations is shown in Table 1. The sensor array was then secured to the top of a weatherproof electronics enclosure for testing.

**Table 1:** Sensor array trace gap spacing between traces and widths of traces.

Sensors	Trace Width (mm)	Spacing (mm)	Gap Width (mm)	Variable Resistor Setting (kΩ)
1, 2, 3, 4	0.41	0.76	0.36	6.0
5, 6	0.41	1.52	1.12	11.0
7, 8	0.18	0.36	0.18	3.0

**Figure 2:**
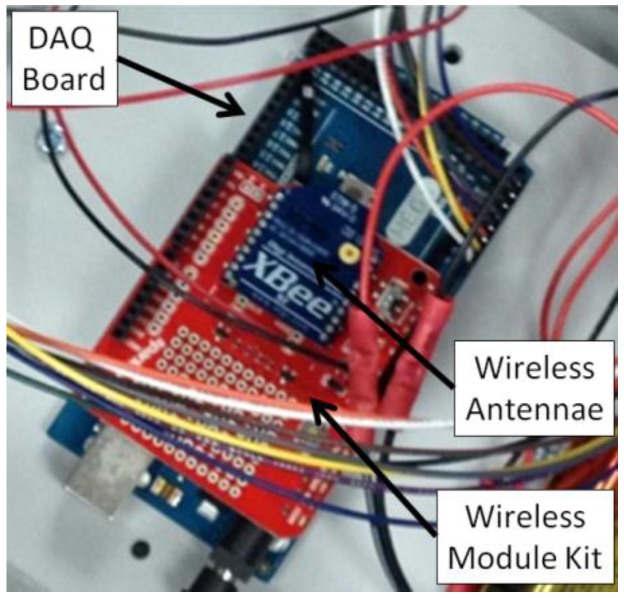
Internal data acquisition and wireless module used for sensor array.

The weatherproof enclosure contained the DAQ board (Arduino MEGA 2560) and the wireless data transfer module (XBee 1 mW trace antenna, Digi International Inc., Minnetonka, MN, USA) as shown in Figure 2. A wireless module kit (KIT-13197, Sparkfun Electronics, Niwot, CO, USA) was used to interface between the wireless transmitter and DAQ board. The same kit contained a module to connect the wireless receiver via a universal serial bus (USB) port on the laptop computer. This allowed for the data from the DAQ board to be recorded on the computer using the serial monitor interface in the software (Arduino v1.0.5-r2).

**Figure 3:**
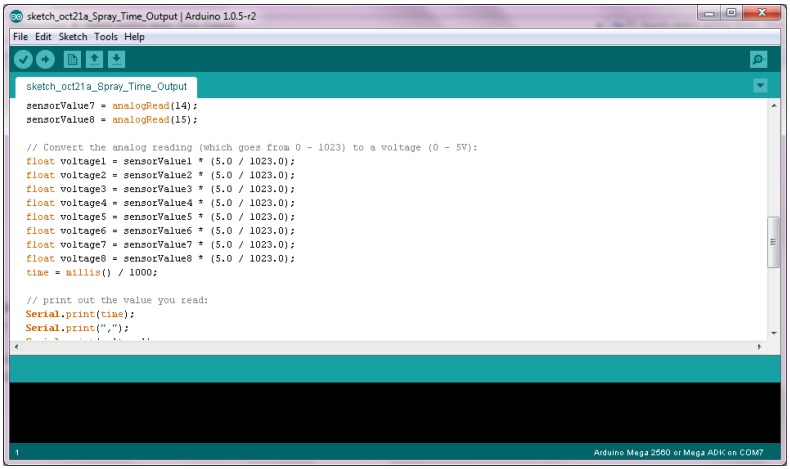
Screenshot showing a portion of the code programmed onto the DAQ board for reading analog voltages and serial string conversion and transmission.

The DAQ board utilized analog inputs to measure the voltage across each sensor (in reference to the common ground) at 1 Hz. The voltage values were then converted to a numeric value and transmitted as a serial data string via the wireless module. The data string transmitted contained cumulative time per test (incremented by 1 s) with voltage output per sensor separated by commas. The resulting data were saved as a .csv file for use with MS Excel. Programming for the DAQ embedded processor was accomplished using an open-source program (Arduino v1.0.5-r2). A screenshot of the code programmed onto the embedded processor is shown in Figure 3.

### 2.2. Test Procedures

#### 2.2.1. Sensor Array Testing with Metered Droplets

The purpose of the metered droplet tests was to evaluate sensor array output consistency and the effects of droplet size, temperature, and placement orientation on sensor output. Tap water was used for the liquid in all of the experiments conducted. For each test, six droplets were placed on each sensor and the output voltage was recorded. The sensor boards were then wiped with a cloth to remove any water and sensor output was checked to ensure that 0 VDC had been achieved prior to the next test. Each test was replicated three times to allow for statistical analysis.

The first variable chosen for this experiment was the droplet size; a pipette was used to dispense 5 and 10 μL droplets on the surface of each sensor. Droplet diameters for the 5 and 10 μL droplets would be approximately 2000 and 2600 μm, respectively based on a spherical shape. While these droplet sizes would generally be very large compared to typical VMD for agricultural spray nozzles, the goal was to verify sensor output sensitivity *versus* liquid applied under controlled conditions. The second variable of the experiment was droplet placement orientation. The droplets were placed horizontally, diagonally, or vertically on the sensors. The droplet orientation test was designed to determine if output voltage from the droplets was affected by placement on the sensor board. The final test variable used was droplet temperature, and tests were repeated using water at temperatures of 1, 25 and 43 °C. Considering three replications of all combinations of droplet size, temperature, and placement orientation, a total of 54 tests were applied to each of the eight sensors for this portion of experiments.

#### 2.2.2. Statistical Analysis

Statistical Analysis Software (SAS v9.4) was used to evaluate the results of the metered droplet tests. A generalized linear model was used to test interactions and nested effects among the independent variables (*i.e.*, treatments). This test was selected because it allowed for both numerical and physical variables to be accounted for when calculating the overall significance level. A fourth independent variable was defined as the sensors themselves within each sensor configuration. The goal was to determine if the output from sensors of a given configuration could be considered consistent. An alpha (α) value of 0.05 was used to test for significant differences at a 95% confidence level. A type III error test was conducted and if the probability was less than 0.05, then the effect of that treatment was considered statistically significant.

#### 2.2.3. Sensor Array Testing with Spray Chamber

The purpose of the spray chamber tests was to evaluate the sensor performance when different application rates and droplet spectra were applied to the sensor array. Three 80° extended-range flat-fan nozzles were selected for testing in the spray chamber; a summary of the test configurations is shown in Table 2. The nozzles were selected because of the potential range of droplet size classifications [9] ranging from fine to coarse at typical operating pressures. All tests utilized tap water at a constant laboratory temperature of 25 °C.

**Table 2:** TeeJet nozzles used in spray chamber testing with corresponding pressure, droplet size classification, application speed and rate.

Nozzle ID Number	Pressure (kPa)	Droplet Size Classification ^†^	Speed (km·h^−1^)	Application Rate ^†^ (L·ha^−1^)
XR8001	207	F	4	96
XR8003	207	M	10	96
XR8005	207	C	10	193

^†^ Droplet size classification and application rate estimated from manufacturer’s nozzle chart (TeeJet, 2015).

The travel speed for nozzles in the spray chamber was limited to speeds between 2 and 10 km·h^−1^. Nozzle operating pressures and speeds were selected to provide for application rates with varying droplet sizes (XR8001 and XR8003 nozzles). The XR8005 nozzle test configuration was selected to provide an example of increased application rate with increase in droplet size. The voltage data from the sensor array were recorded for several minutes after the nozzle passed over the system. Sensor output values *versus* time were then plotted to observe results from the different treatments. Differences in output from the three sensor configurations were of particular importance to determine any changes based on nozzle operating conditions.

## 3. Results and Discussion

### 3.1. Sensor Array Testing with Metered Droplets

Output voltages from the sensors indicated that there were no significant differences among the sensors when considering droplet placement orientation on the sensor surface. In addition, each sensor output was considered consistent (*i.e.*, not significantly different) among the three sensor configurations tested. This result was desirable as it showed that these sensors could be a viable option to return consistent voltage values during normal use.

The effects of temperature on sensor output voltage varied. As the water temperature increased, there was a slight, however significant; change in the output voltages for all sensors when 10 μL droplets were placed on the surface. This trend was not noticed for the sensors when 5 μL droplets were used. These data suggest that an additional temperature sensor may be required for in-field use. However, if water were kept at constant temperature in a laboratory setting, for example, such a sensor would not likely be necessary. The results from the metered droplet size tests showed that droplet size was significantly different (*p* ≤ 0.05). This result was expected as increased water volume on the sensors should have resulted in higher output voltage.

### 3.2. Sensor Array Testing with Spray Chamber

**Figure 4:**
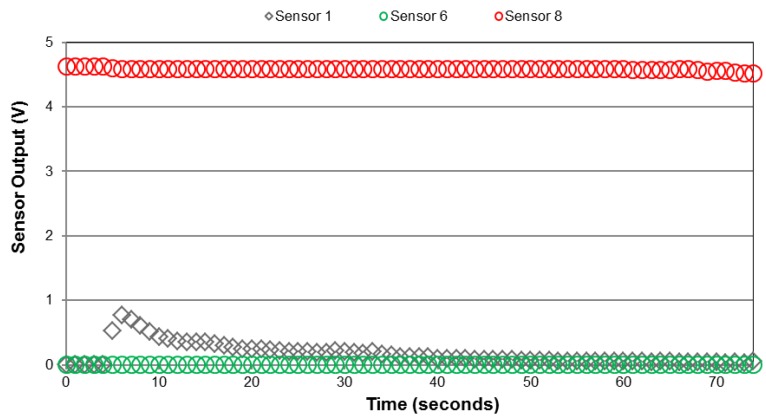
Sensor output voltage *versus* time from XR 8001 nozzle at 207 kPa (Fine droplet spectra classification).

Voltage output from Sensors 1, 6, and 8 (shown as examples) from the spray chamber tests using the XR 8001 nozzle at 207 kPa are shown *versus* time in Figure 4. At that pressure, this nozzle was expected to provide Fine droplets based on the datasheet [9] provided by the manufacturer. The output from these sensors provides insight into how this array might be used to distinguish among different droplet classifications. The smallest sensor trace/gap width configuration (Sensors 7 and 8) recorded nearly 5 VDC immediately after application which indicated that the sensor output was saturated. The next largest sensor configuration (Sensors 1 through 4) indicated a brief spike in voltage output followed by a gradual decline in sensor output. The source of the spike was unknown but dissipated within several seconds after spray exposure. However the cumulative trend in these data was similar to results noted by Salyani and Serdynski (1990), the effect was hypothesized to be due to electrochemical processes on the sensor surface. The largest sensor configuration (Sensors 5 and 6) indicated no response to the application of water from the XR 8001 at 207 kPa. This was likely due to smaller particles that could not bridge between the sensor trace gaps. While not completely conclusive, these results to indicate that the smallest sensors could detect surface application while the larger sensors would be required to distinguish among different particle sizes.

**Figure 5:**
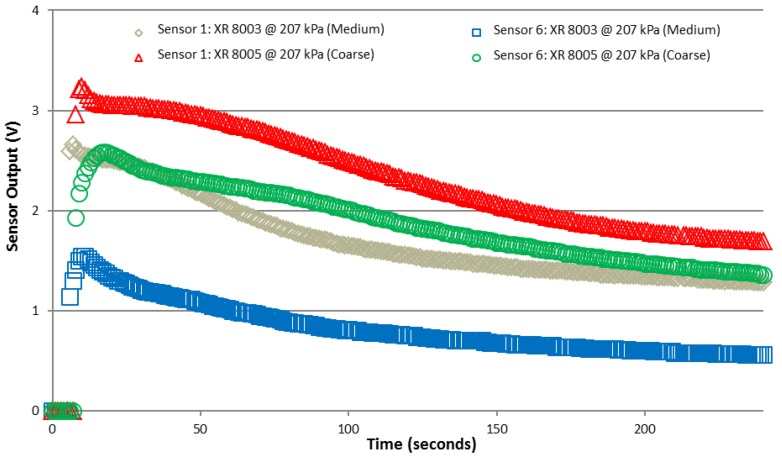
Sensors 1 and 6 voltage output from XR 8003 and XR 8005 nozzles at 207 kPa operated at constant speed in spray chamber.

Further tests using larger nozzles (XR 8003 and XR 8005) showed that increased application rates and droplet sizes increased sensor output voltage for the two larger sensors from the array (Figure 4). It should be noted that output from Sensors 7 and 8 was again saturated at nearly 5 VDC (not shown in Figure 5). Output from the two larger sensor configurations provided three valuable pieces of information. First, in comparison to the data in Figure 4, Sensors 5 and 6 were able to register application to the sensor surface. This suggested that at higher application rates and droplet sizes the larger configuration was able to register spray deposition. The magnitude of the output voltage increased for Sensors 1 through 4 as the droplet sizes increased as well. Note that due to travel speeds and operating pressures summarized in Table 2, the application rates should have been similar in this case. Secondly, the output from Sensors 1 through 4 was slightly higher as application rates increased compared to the largest sensor configuration (*i.e.*, Sensors 5 and 6). This was expected as more trace material was present for conducting the electrical signal for these sensors. Finally, for the two largest sensor configurations shown in Figure 5, output voltage increased in both cases with the increase in application rates and droplet sizes. There was a slight difference in output decay rate between the two representative sensor datasets when observing the different nozzles shown in Figure 4. Sensor output declined at a slower rate which was most likely due to the increased water deposited from the larger nozzles (compared to the XR 8003) at a constant pressure.

## 4. Conclusions

A wireless electronic sensor array was developed and preliminary tests were conducted to evaluate the feasibility of the system for estimating application rates and droplet size classifications from agricultural spray nozzles. Metered droplets were placed on the sensor array surface to evaluate any effects from droplet size, temperature, or droplet orientation on the sensor surface. Statistical analysis determined that droplet orientation had no effect on sensor output. Droplet temperature had mixed effects; in one case an increase in temperature did result in higher output voltages. It was concluded that under field conditions, monitoring sensor temperature may be important; however, in a lab environment, droplet temperature could potentially be controller. There was a significant increase in output voltage from all sensors when the metered droplets increased in size from 5 μL to 10 μL. Further testing in a spray chamber provided preliminary results on the output from the different sensor configurations. Output voltages from the different sensor sizes indicated that variations in application rate and droplet sizes could be seen using multiple sensor configurations. Further tests are needed (and pending) to separate out the effects of application rate *versus* droplet sizes from agricultural hydraulic nozzles.
